# Supplementary material for: Entangling Mobility and Interactions in Social Media
Source: PLoS One. 2014 Mar 20;9(3):e92196. doi: 10.1371/journal.pone.0092196 (PMC3961345; doi:10.1371/journal.pone.0092196)
Supplement: Table S1 — The contribution of each of the properties to the total error of the TF model. Value of the error per property X at the minimum of the total error Err for Twitter for the three considered countries. (PDF) [file pone.0092196.s009.pdf]

	P_{l}	$P(k)$	$R(d)$	$J_{\mathrm{f}}(d)$	$C(d)$	$P(D)$	N_{c}	C_{avg}	Err
US	0.60	0.35	0.17	0.33	0.56	0.41	0.01	0.05	0.30
UK	0.20	0.43	0.15	0.25	0.25	0.06	0.02	0.34	0.18
DE	0.56	0.60	0.53	0.36	0.21	0.36	0.56	0.17	0.39
