# Supplementary material for: Entangling Mobility and Interactions in Social Media
Source: PLoS One. 2014 Mar 20;9(3):e92196. doi: 10.1371/journal.pone.0092196 (PMC3961345; doi:10.1371/journal.pone.0092196)
Supplement: Appendix S1 — Variants of the TF model. In this appendix, we consider several variants of the TF model and the L model and evaluate their results. Appendix S1 contains Figures S9 and S10. (PDF) [file pone.0092196.s010.pdf]

Appendix S1: Variants of the TF model

P.A. Grabowicz, J.J. Ramasco, B. Gonçalves & V.M. Eguíluz

In this section, we consider several variants of the TF model and the L model and evaluate their results. We describe a total of 36 variants marked with different colors in the tables in Figures S9 and S10. For each variant we explore the space of the parameters p_v and p_c. We run the models for p_v from the set $\{0, 0.1, 0.2, 0.3, 0.4, 0.5, 0.6, 0.7\}$ and p_c from $\{0, 0.0003, 0.001, 0.003, 0.01, 0.03\}$, yielding in total 48 parameter combinations. For each of the model variants, we find the parameters that minimize the fitting error E. We plot its value in Figures S9 and S10. In the following paragraphs, we describe in detail each of the variants and its results.

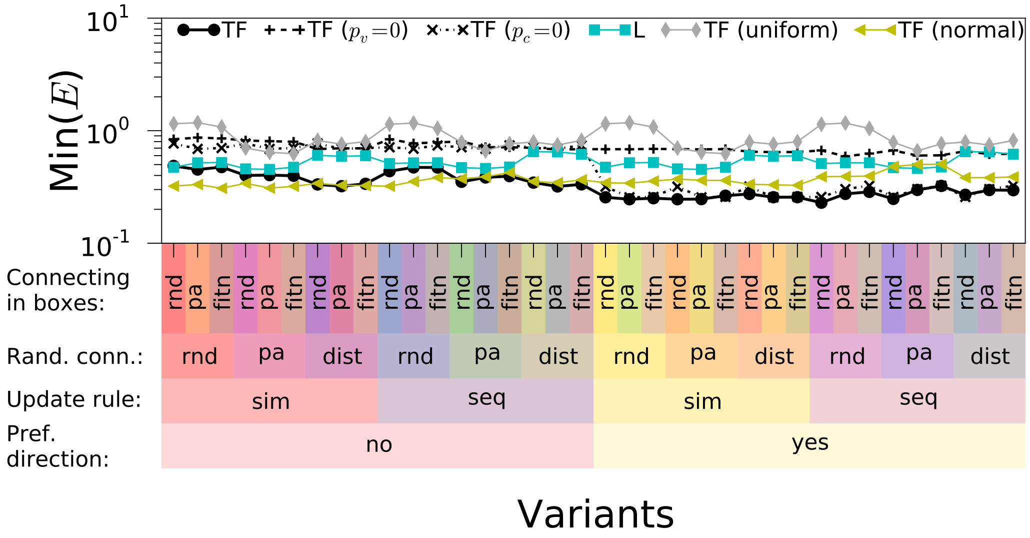

Figure S9: **The model variants.** Values of the fitting error E for the UK for the variants of the following models: the TF model, the TF model with $p_v = 0$, the TF model with $p_c = 0$, the L model and the TF model with uniformly or normally distributed jumps. The default variant described in the manuscript is marked with the the red rectangle.

First, we modify the jump size distribution to understand its impact on the geo-social properties. We consider the following cases: the default power-law jumps with exponent 1.55, a minimal jump length of 1 km and a cutoff at 100 km, as in [1] (the TF model), uniformly distributed random jumps up to 100 km (TF-uniform), normally distributed jumps with standard deviation of 1 km (TF-normal), and no jumps to new locations at all (the L model). We plot the minimal fitting error

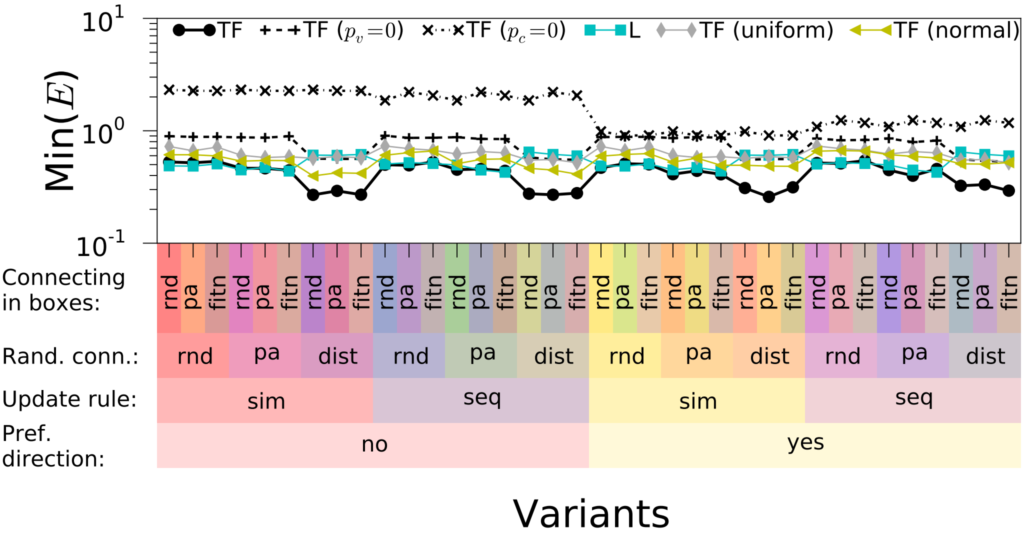

Figure S10: **The model variants.** Values of the fitting error E for Germany for the variants of the models as in Figure S9.

of these cases in Figures S9 and S10 using different colors of the curves. The default power-law jumps show the best results with the lowest error for most of the variants. The normal distribution and the L model tend to perform considerably worse. The highly unrealistic uniform jumps understandingly provide the worst results and the highest error values for almost all variants.

To assess the role of friend visits and random connections we turn on and off these two components by setting to zero the corresponding parameters p_v and p_c. We plot the results in Figures S9 and S10 with dashed and dotted lines. We observe significantly higher error values whenever one of these two components is turned off, for most of the model's variants, what demonstrates their importance for the TF model.

To prevent users from spreading into inhabited regions, we include in the TF model an angular preference for the jumps. Namely, the direction of each jump is chosen randomly with a probability proportional to the number of inhabitants present at the destination. Note that this does not affect in any way the length of the jumps, which is drawn independently beforehand. To estimate the population of the target area, we use the gridded population of the world[1]. To test how this angular preference impacts the results, we consider a variation of the models without it and compare the results. The two variants are included in the lowest row of the table in Figures S9 and S10. They show almost no difference in the error values for the Germany, although a systematic difference exists for the UK; the error of the variant with direction preference is consistently lower in the case of that country. The presence of the sea around the UK introduces a distorting factor for the TF model. Without the directional preference the agents freely spread over the sea independently on the geographical shape of the country, leading to unrealistic results.

[1]The Gridded Population of the World and The Global Rural-Urban Mapping Projects, Socioeconomic Data and Applications Center of Columbia University, http://sedac.ciesin.columbia.edu/gpw.

Agents' traveling and link creation can be realized in the simulation in various update orders. By default, at each time step, each agent first moves, next connects to other random agents, and then connects locally; the following agent performs the same actions in the same order, etc. We call this method sequential ("seq"). In an alternative update rule, which we call simultaneous ("sim"), first all the agents move, then all of them create random connections, and finally all of them create connections locally. The two update rules are included in the second row, counting from the bottom, of the table in Figures S9 and S10. The update rules have little impact on the final networks resulting from the simulation.

In the TF and L models, the agents create with probability p_c random connections. These links can be created in different ways; we consider three variants. First, each agent chooses another agent uniformly at random, what constitutes the default mechanism ("rnd"). Second, each agent randomly picks another node with probability proportional to the current degree of the node, which corresponds to the preferential attachment mechanism ("pa"). Third, the agent draws another node with probability decaying as a power-law of the distance between the two agents ("dist"), with its exponent equal to 1.4 and the minimal distance of 0.1 km. The type of random connecting mechanism used is listed in the third row, counting from the bottom, of the table in Figures S9 and S10. In some cases, e.g., for Germany, the distant-dependent probability of creating a random link provides better results than the other variants.

We consider similar variants for the connections formed inside spatial boxes, which are created with the probability p. The agents can connect uniformly at random ("rnd"), with a preference for high-degree nodes ("pa"), or a preference toward the nodes with high intrinsic fitness ("fitn"). The fitness of the nodes is drawn from a power-law distribution with an exponent of 1.5, which roughly corresponds to the distribution of the growth rates reported in [2]. These variants are implemented in the following way. First, we note that the number of connections created by the agent is a result of a binomial process with probability p and the number of trials is equal to the number of agents that currently stay in the given spatial box. The expected number of links created in such binomial process is known, therefore, an equivalent number of connections can be created with one of the two mentioned preferential processes. The type of connecting mechanism applied in the spatial boxes is listed in the top row of the table in Figures S9 and S10. There is no consistent difference in the error values between these variants. Thus, the connecting mechanism applied in the spatial boxes has little impact on the results.

We conclude that the main components of the TF models are crucial to reproduce the structure and geography of the social networks. These components include the mobility model, friend visits and random connections. The power-law mobility model tends to produce the best results. The angular preference of travels is important for countries whose geography is strongly restrained, e.g., by sea. Other modifications to the model have low or no consistent impact on the results, with the exception of the distance dependent random connections, which in certain cases consistently influence the results.

References

[1] Song C, Koren T, Wang P, Barabási AL (2010) Modelling the scaling properties of human mobility. Nature Physics 6: 818–823.

[2] Grabowicz PA, Eguíluz VM (2012) Heterogeneity shapes groups growth in social online communities. EPL (Europhysics Letters) 97: 28002.
